# Postprandial Hyperglycemia Is Associated With White Matter Hyperintensity and Brain Atrophy in Older Patients With Type 2 Diabetes Mellitus

**DOI:** 10.3389/fnagi.2018.00273

**Published:** 2018-09-12

**Authors:** Noriko Ogama, Takashi Sakurai, Shuji Kawashima, Takahisa Tanikawa, Haruhiko Tokuda, Shosuke Satake, Hisayuki Miura, Atsuya Shimizu, Manabu Kokubo, Shumpei Niida, Kenji Toba, Hiroyuki Umegaki, Masafumi Kuzuya

**Affiliations:** ^1^Center for Comprehensive Care and Research on Memory Disorders, National Center for Geriatrics and Gerontology, Obu, Japan; ^2^Department of Geriatric Medicine, National Center for Geriatrics and Gerontology, Obu, Japan; ^3^Department of Community Healthcare and Geriatrics, Nagoya University Graduate School of Medicine, Nagoya, Japan; ^4^Department of Cognition and Behavior Science, Nagoya University Graduate School of Medicine, Nagoya, Japan; ^5^Department of Diabetes and Endocrinology, National Center for Geriatrics and Gerontology, Obu, Japan; ^6^Department of Clinical Laboratory, National Center for Geriatrics and Gerontology, Obu, Japan; ^7^Medical Genome Center, National Center for Geriatrics and Gerontology, Obu, Japan; ^8^Department of Home Care Coordinators, National Center for Geriatrics and Gerontology, Obu, Japan; ^9^Department of Cardiology, National Center for Geriatrics and Gerontology, Obu, Japan; ^10^Institutes of Innovation for Future Society, Nagoya University, Nagoya, Japan

**Keywords:** Alzheimer’s disease, brain atrophy, diabetes mellitus, postprandial hyperglycemia, white matter hyperintensity

## Abstract

Type 2 diabetes mellitus is associated with neurodegeneration and cerebrovascular disease. However, the precise mechanism underlying the effects of glucose management on brain abnormalities is not fully understood. The differential impacts of glucose alteration on brain changes in patients with and without cognitive impairment are also unclear. This cross-sectional study included 57 older type 2 diabetes patients with a diagnosis of Alzheimer’s disease (AD) or normal cognition (NC). We examined the effects of hypoglycemia, postprandial hyperglycemia and glucose fluctuations on regional white matter hyperintensity (WMH) and brain atrophy among these patients. In a multiple regression analysis, postprandial hyperglycemia was independently associated with frontal WMH in the AD patients. In addition, postprandial hyperglycemia was significantly associated with brain atrophy, regardless of the presence of cognitive decline. Altogether, our findings indicate that postprandial hyperglycemia is associated with WMH in AD patients but not NC patients, which suggests that AD patients are more susceptible to postprandial hyperglycemia associated with WMH.

## Introduction

Increasing evidence shows that type 2 diabetes mellitus is associated with neurodegeneration and cerebral vascular disease (Arnold et al., 2018). The presence of diabetes is a significant risk factor for the incidence of Alzheimer’s disease (AD) and vascular dementia (VaD) in the elderly (Ohara et al., 2011). Although the mechanism underlying its relationship with these phenomena is not fully understood, clinical features such as cognitive deficits, large- and small-vessel disease, cerebral atrophy, hypometabolism, and impaired insulin activation in the brain, overlap between type 2 diabetes and AD (Arnold et al., 2018).

White matter hyperintensity (WMH), one of the cerebral small vessel diseases, presents as hyperintense areas on T2-weighted and fluid-attenuated inversion recovery (FLAIR) images and as isointense or hypointense areas on T1-weighted images on magnetic resonance (MR) imaging. WMH is a common finding in the aging brain and plays pivotal roles in several geriatric syndromes, including cognitive impairment, gait disturbances and functional decline of the elderly (Akisaki et al., 2006; Ogama et al., 2014, 2017; Tomimoto, 2015; Tamura et al., 2017). Thus, preventive strategies capable of delaying WMH progression should be identified to successfully manage older people with diabetes.

Currently, age and hypertension are recognized as consistent risk factors for WMH (Tomimoto, 2015). However, whether diabetes is independently associated with WMH has not been clarified. Several studies have reported that WMH volume is increased in patients with diabetes compared with non-diabetic subjects (Novak et al., 2006; Raffield et al., 2016); however, other studies did not observe this finding (Falvey et al., 2013; Moran et al., 2013). Some studies have focused on the association between HbA1c or fasting plasma glucose levels and WMH; however, the results of these studies were inconsistent (Bryan et al., 2014; Exalto et al., 2014; Geijselaers et al., 2015; Raffield et al., 2016; Schneider et al., 2017). More recently, an association between fluctuations in HbA1c levels and WMH was reported in diabetes patients who carry the apolipoprotein E4 allele (Livny et al., 2016), which is a strong genetic risk factor for AD. Additionally, apolipoprotein E4 carriers who are elderly and have diabetes or higher HbA1c levels exhibited greater WMH progression than other individuals (Cox et al., 2017). These findings suggest that patients with diabetes who have experienced AD-related pathological changes in the brain may be more susceptible to metabolic stress than other patients.

Several imaging studies have reported that glucose alterations are associated with regional brain changes and dysfunctions. Diabetes patients with higher HbA1c levels exhibit regional brain atrophy, including as affected by the AD signature (Schneider et al., 2017), and high insulin resistance was significantly associated with lower cerebral perfusion in frontal and temporal regions (Hoscheidt et al., 2017). However, regarding hypoglycemia, it remains controversial whether a hypoglycemic event is associated with brain dysfunction. Patients with symptomatic severe hypoglycemia exhibit similar patterns of brain atrophy and white matter abnormalities as those without a hypoglycemic episode (Zhang et al., 2014). Conversely, another study reported that patients with prolonged hypoglycemic episodes show signal abnormalities in temporal and occipital lobes (Boeve et al., 1995). Importantly, hypoglycemic episodes in older diabetes patients are unlikely to be reported due to mild or absent symptoms (Abdelhafiz et al., 2015). Thus, to investigate the effects of hypoglycemia on brain abnormalities in elderly patients with diabetes, it is necessary to carefully investigate mild hypoglycemia that does not appear as a typical symptom and is not reported by patients.

To the best of our knowledge, no studies have examined differences in the effects of glucose alteration on brain abnormalities in diabetic patients with and without cognitive decline. The aim of the present study was to clarify the effects of glucose alteration on regional WMH and brain atrophy in older type 2 diabetes patients who have a diagnosis of AD or normal cognition (NC). In particular, we focused on the effects of hypoglycemia, postprandial hyperglycemia, and glucose fluctuations on regional WMH and brain atrophy among these patients. We measured daily glucose profiles using self-monitored blood glucose (SMBG) and also measured HbA1c levels. We hypothesized that abnormal glucose profiles in diabetes patients with AD are more likely to be associated with brain structural changes compared with NC patients.

## Materials and Methods

### Participants

This study was approved by the Ethics Review Board of Japan’s National Center for Geriatrics and Gerontology (NCGG). All candidate subjects and their caregivers provided written informed consent to participate in the study prior to enrolling. We included 57 outpatients (AD: *n* = 23; NC: *n* = 34) who visited our hospital from 2014 to 2015 in the study. The diagnosis of probable or possible AD was based on the findings of the National Institute on Aging/Alzheimer’s Association workgroups on the diagnostic guidelines for Alzheimer’s disease (McKhann et al., 2011). The following patients were included in the study: (a) outpatients with type 2 diabetes who were treated with antidiabetic agents, (b) patients aged 65 years or older, (c) patients living at their houses, (d) patients with a Mini-Mental State Examination (MMSE) score ≥ 10 for AD and (e) patients with family members or caregivers who assist with SMBG. The following patients were excluded from the study: (a) patients with type 1 diabetes, (b) patients with cortical lesions on MR imaging, (c) patients with neurological disorders other than AD and (d) patients with severe conditions, such as cardiac failure, renal disorder, or liver dysfunction.

### Assessment of Clinical Parameters and Diabetic Complications

The clinical data and blood samples used in this study were distributed from the NCGG Biobank, which collects and stores biological material and associated clinical information for biomedical research. Information regarding patient diagnoses, clinical information and medications was obtained from patient clinical charts. Global cognitive function was assessed by the MMSE (Folstein et al., 1975).

The incidence of diabetic complications was determined based on the rates at which neuropathy, retinopathy and nephropathy co-existed with diabetes (Akisaki et al., 2006; Sakurai et al., 2012). Diabetic neuropathy was defined as either the loss of the Achilles tendon reflex or the presence neuropathic symptoms, such as paresthesia. Diabetic retinopathy was assessed by fundoscopy of dilated pupils, which was performed by experienced ophthalmologists. Diabetic nephropathy was assessed by measurements of the urinary albumin-to-creatinine ratio (ACR) and urinary protein concentrations, and nephropathy was defined as an ACR > 300 μg/mg or a urinary protein concentration > 30 mg/dL. The following biochemical indices were assessed in the study: HbA1c, triglycerides, total cholesterol, high-density cholesterol, low-density cholesterol, the estimated glomerular filtration rate, albumin and urinary albumin. HbA1c levels are expressed in National Glycohemoglobin Standardization Program units.

Daily glycemic levels were evaluated using SMBG (MS-FR201B; Terumo Corp., Tokyo, Japan) and were measured at 5 time points per day (5 AM, before breakfast, 2 h after breakfast, before lunch, and before dinner) on 8 separate days during a 2-month period. A previous study using continuous glucose monitoring (CGM) showed that glucose levels usually nadir early in the morning and before each meal and that postprandial glucose levels were likely to peak at 2 h after breakfast in patients with type 2 diabetes (Monnier et al., 2007); therefore, we measured blood glucose levels at these time points. When patients with AD used SMBG, their families assisted with the measurements. Hypoglycemia was defined as a glucose level ≤ 70 mg/dL (Workgroup on Hypoglycemia, American Diabetes Association et al., 2005), and the presence or absence of hypoglycemic symptoms was reported at every SMBG measuring point. Glycemic fluctuations were calculated as diurnal ranges from minimum glucose levels to maximum glucose levels.

### Evaluation of WMH and Brain Atrophy

All participants underwent T1- and T2-weighted and FLAIR MR imaging with 1.5T MR scanners (Siemens Avanto, Munich, Germany; or Philips Ingenia, Eindhoven, The Netherlands). WMH and brain atrophy were analyzed by an automatic segmentation application (SNIPER, Software for Neuro-Image Processing in Experimental Research: Department of Radiology, Leiden University Medical Center, The Netherlands) (Admiraal-Behloul et al., 2005). WMH was evaluated in the frontal, temporal, occipital and parietal lobes. Global brain atrophy was calculated as the parenchyma (PAR) volume, which is determined by subtracting the cerebrospinal fluid volume from the intracranial (IC) volume. The PAR volume corresponds to the sum of the total gray and white matter volumes. WMH and brain atrophy parameters were divided by the IC volume to adjust for the size of each patient’s brain. Further details regarding the MR scanning protocol and the automatic segmentation application are provided elsewhere (Admiraal-Behloul et al., 2005; Ogama et al., 2016).

To avoid misclassification of WMH in SNIPER, WMH segmentation was replicated using Lesion Segmentation Tool (LST) in Statistical Parametric Mapping 12 (Schmidt et al., 2012). LST is an automated segmentation approach for quantifying whole-brain WMH volume, and shows high agreement with manual tracing of WMH in FLAIR images (Schmidt et al., 2012).

### Statistical Analysis

All analyses were performed using the Japanese version of SPSS for Windows version 22.0 (IBM Corp., Armonk, NY, United States). Differences between the clinical profiles of the AD and NC groups were examined using the *t*-test (for parametric variables), Mann–Whitney *U* test (for non-parametric variables), and chi-squared test or Fisher’s exact test (for categorical variables). To assess the association between each daily glucose index and WMH and brain atrophy, we performed multiple regression analysis. The dependent variables were WMH and brain atrophy, and the independent variables were the daily glucose indices. To identify the independent risk factors for WMH and brain atrophy, we performed multiple regression analysis with step-wise methods. Daily glucose indices that were in association in the previous analysis (*p* < 0.10) were entered into this analysis as independent variables. Age, hypertension and HbA1c were entered into the analysis as adjustment variables for WMH, and age and HbA1c were entered into the analysis as possible confounders for brain atrophy. Statistical significance was set at *p* < 0.05.

## Results

### Clinical Features of the Study Participants

The clinical profiles of the study participants are shown in **Table 1**. The mean (±SD) age was 75.6 ± 5.8 years in the AD group and 74.1 ± 4.6 years in the NC group, respectively. The MMSE score was significantly lower in the AD group than in the NC group. There were no significant differences in the rates of diabetic comorbidities and antidiabetic agent use between the two groups. Although average HbA1c and serum triglyceride levels were higher in the AD group than NC group, the levels of the other metabolic and nutritional markers analyzed herein did not differ between the two groups. These results indicate that the diabetes statuses of the AD and NC groups were largely comparable.

**Table 1:** Clinical characteristics of the study participants (*n* = 57).

	AD (*n* = 23)	NC (*n* = 34)	*p*-value
Age, years	75.6 (5.8)	74.1 (4.6)	0.289
Male, n (%)	12 (52.2)	19 (55.9)	0.783
Education, years	11.3 (2.6)	11.5 (2.6)	0.519
Duration of diabetes, years	14.2 (10.4)	14.4 (9.3)	0.665
Mini-Mental State Examination	20.4 (4.5)	27.4 (2.1)	<0.001
Body mass index, kg/m^2^	23.5 (2.8)	24.2 (2.7)	0.347
History of hypertension, n (%)	17 (73.9)	27 (79.4)	0.627
History of coronary artery disease, n (%)	5 (21.7)	8 (23.5)	0.874
Comorbidities of diabetes, n (%)			
Diabetic neuropathy	16 (69.6)	21 (61.8)	0.545
Diabetic retinopathy	3 (13.0)	11 (32.4)	0.097
Diabetic nephropathy	9 (39.1)	9 (26.5)	0.313
Antidiabetic agents, n (%)			
Biguanide	6 (26.1)	10 (29.4)	0.784
Thiazolidine	5 (21.7)	2 (5.9)	0.106
DPP4 inhibitor	17 (73.9)	23 (67.6)	0.612
Sulfonylurea	12 (52.2)	20 (58.8)	0.620
Insulin secretion promoter	2 (8.7)	0 (0.0)	0.159
α-glucosidase inhibitor	2 (8.7)	9 (26.5)	0.170
Insulin	5 (21.7)	6 (17.6)	0.742
GLP-1 receptor agonists	1 (4.3)	1 (2.9)	1.000
Biochemical examination			
HbA1c, %	7.4 (0.7)	7.1 (0.5)	0.044
Triglycerides, mg/dL	170.0 (81.1)	120.0 (58.3)	0.019
Total cholesterol, mg/dL	188.1 (43.3)	190.7 (42.8)	0.968
HDL cholesterol, mg/dL	52.4 (15.4)	55.6 (13.6)	0.425
LDL cholesterol, mg/dL	102.9 (37.9)	110.4 (36.2)	0.384
eGFR, mL/min/1.73 m^2^	65.2 (17.9)	62.7 (18.6)	0.623
Albumin, g/dL	4.2 (0.4)	4.4 (0.3)	0.105
UACR, mg/gCr	116.6 (213.9)	158.1 (348.2)	0.362
Self-monitoring of blood glucose			
5 AM, mg/dL	112.5 (21.8)	120.1 (25.1)	0.337
Before breakfast, mg/dL	119.4 (21.2)	126.9 (23.3)	0.222
2 h after breakfast, mg/dL	183.2 (38.7)	178.6 (28.8)	0.833
Before lunch, mg/dL	139.4 (44.3)	118.2 (26.9)	0.053
Before dinner, mg/dL	139.1 (37.6)	133.9 (19.8)	0.814
Fluctuation, mg/dL	96.8 (32.7)	86.9 (24.8)	0.200
Frequency of hypoglycemia^∗^	0.61 (1.6)	0.62 (1.1)	0.579
MR imaging			
IC, mL	1458.2 (130.4)	1400.9 (141.1)	0.127
PAR, mL, % of IC	1113.7 (125.4), 76.3%	1117.8 (140.2), 79.7%	0.003
WMH, mL, % of IC	16.6 (13.0), 1.14%	11.0 (12.8), 0.79%	0.029
Frontal lobe, mL, % of IC	10.9 (9.4), 0.76%	6.1 (6.1), 0.44%	0.010
Temporal lobe, mL, % of IC	1.0 (1.2), 0.07%	0.9 (1.5), 0.06%	0.216
Occipital lobe, mL, % of IC	0.5 (0.4), 0.03%	0.4 (0.5), 0.03%	0.162
Parietal lobe, mL, % of IC	4.1 (3.9), 0.28%	3.5 (5.2), 0.26%	0.126
Periventricular area, mL, % of IC	15.4 (13.1), 1.06%	10.3 (12.7), 0.74%	0.029
Deep subcortical areas, mL, % of IC	1.2 (1.3), 0.08%	0.7 (0.9), 0.05%	0.216

*Data are presented as the mean (standard deviation) or as numbers and percentages unless otherwise indicated. ^∗^Indicated per-patient averages at the measuring points during the study period. AD, Alzheimer’s disease; DPP4, dipeptidyl peptidase 4; eGFR, estimated glomerular filtration rate; GLP-1, glucagon-like peptide-1; HDL cholesterol, high-density lipoprotein cholesterol; IC, intracranial; LDL, low-density lipoprotein cholesterol; MR, magnetic resonance; NC, normal cognition; PAR, parenchyma; UACR, urine albumin-to-creatinine ratio; WMH, white matter hyperintensity.*

The average blood glucose levels at the 5 SMBG measuring points used during each of the 8 days are shown in **Table 1**. Mean pre-lunch glucose levels tended to be higher in the AD group than NC group, but there were no significant differences in any daily glucose indices between the two groups. Hypoglycemia (blood glucose range: 43 mg/dL – 70 mg/dL) was observed at 14 measuring points (1.5% of all measuring points) in the AD group and 21 measuring points (blood glucose range: 45 mg/dL – 70 mg/dL, 1.5% of all measuring points) in the NC group. Severe hypoglycemic symptoms and events were not reported in this study. Typical hypoglycemic symptoms, such as palpitations and hand tremors, were observed in one individual in the NC group whose glucose level was 45 mg/dL. Hypoglycemia-related symptoms, including nausea, dizziness, light-headedness, blurred vision and fatigue, were reported at 4 measuring points (0.4%) in the AD group and 3 measuring points (0.2%) in the NC group during the 2-month period.

**FIGURE 1:**
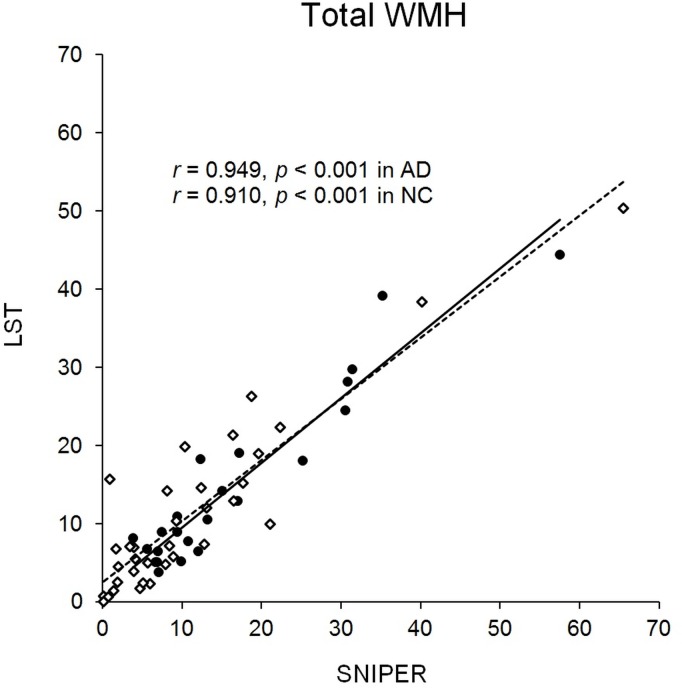
Distribution of total WMH. Scatterplot showing distribution of total WMH (ml) in SNIPER and LST. Black dots and the solid line represent AD. White dots and the dashed line represent NC. AD, Alzheimer’s disease; LST, Lesion Segmentation Tool; NC, normal cognition; SNIPER, Software for Neuro-Image Processing in Experimental Research; WMH, white matter hyperintensity.

Total WMH volume of all subjects was 13.3 ± 13.1 ml in SNIPER and 12.7 ± 11.2 ml in LST. Total WMH volume in each application program showed high correlation (**Figure 1**), suggesting that the method of measurement used for SNIPER was reliable. MR imaging evaluated by SNIPER showed that the AD group had a significantly greater WMH volume than the NC group, particularly in the frontal lobe. In addition, patients in the AD group had more severe brain atrophy than patients in the NC group. Because the deep white matter hyperintensity (DWMH) volume was markedly smaller than the periventricular hyperintensity (PVH) volume in both patient groups, we combined the PVH and DWMH volumes into the WMH volume in all brain regions in subsequent analyses.

### Associations Between Glucose Indices and WMH and Brain Atrophy

The relationships between several glucose indices and MR imaging parameters are shown in **Table 2**. Because age and hypertension are consistent risk factors for WMH, these variables were entered into the analysis as adjustment variables for WMH, while age was entered as an adjustment variable for brain atrophy. Glucose levels at 2 h after breakfast and daily fluctuations in blood glucose levels were associated with WMH in the frontal lobe in the AD group. Glucose levels at 2 h after breakfast and fluctuations in glucose levels tended to be inversely associated with PAR volume. The frequency of hypoglycemia (glucose level ≤ 70 mg/dL) was not correlated with WMH or brain atrophy. The effect of mild hypoglycemia (glucose level ≤ 80 mg/dL or ≤90 mg/dL) on brain structural changes was also examined in this study. Mild hypoglycemia defined as a glucose level ≤ 80 mg/dL was observed at 5.1% of all SMBG measuring points, and mild hypoglycemia defined as a low glucose level ≤ 90 mg/dL was observed at 14.0% of all SMBG measuring points in the AD group. However, there were no associations between the frequency of mild hypoglycemia and MR imaging parameters.

**Table 2:** Associations between glucose indices and WMH and brain atrophy.

	White matter hyperintensity										PAR	
	Total		Frontal lobe		Temporal lobe		Occipital lobe		Parietal lobe			
	*β*	*p*-value	*β*	*p*-value	*β*	*p*-value	*β*	*p*-value	*β*	*p*-value	*β*	*p*-value
**AD subjects**												
5 AM	−0.020	0.929	−0.036	0.877	−0.004	0.987	0.061	0.771	0.014	0.953	−0.357	0.099
Before breakfast	0.098	0.670	0.095	0.680	0.029	0.899	0.036	0.864	0.076	0.744	−0.223	0.313
2 h after breakfast	0.397	0.099	0.521	0.027	−0.131	0.596	−0.112	0.626	0.055	0.827	−0.438	0.061
Before lunch	0.074	0.776	0.088	0.737	0.089	0.731	0.208	0.384	−0.032	0.903	−0.396	0.109
Before dinner	0.243	0.289	0.203	0.381	0.145	0.528	0.377	0.067	0.239	0.303	−0.326	0.139
Fluctuation	0.385	0.086	0.502	0.022	−0.167	0.468	0.000	0.999	0.068	0.774	−0.419	0.051
Frequency of hypoglycemia	0.042	0.863	0.071	0.773	−0.096	0.691	−0.051	0.822	−0.008	0.974	−0.218	0.346
**NC subjects**												
5 AM	−0.124	0.510	−0.091	0.633	−0.177	0.342	−0.109	0.548	−0.136	0.474	−0.160	0.328
Before breakfast	−0.015	0.939	0.010	0.958	−0.068	0.724	−0.097	0.605	−0.019	0.924	−0.155	0.347
2 h after breakfast	−0.217	0.223	−0.230	0.197	−0.142	0.426	−0.074	0.667	−0.214	0.234	−0.470	0.001
Before lunch	−0.098	0.588	−0.069	0.705	−0.135	0.453	−0.193	0.266	−0.101	0.581	−0.095	0.546
Before dinner	−0.092	0.610	−0.070	0.700	−0.107	0.547	−0.033	0.848	−0.107	0.556	−0.087	0.574
Fluctuation	−0.161	0.370	−0.182	0.311	−0.053	0.768	−0.052	0.766	−0.161	0.375	−0.393	0.007
Frequency of hypoglycemia	0.229	0.222	0.146	0.443	0.369	0.042	0.162	0.371	0.270	0.152	0.011	0.942

*Multiple regression analysis was performed with the forced-entry method. The dependent variables were the MR imaging parameters. The independent variables were the daily glucose indices. Age and hypertension were entered into the analysis as classical confounders for WMH. Age was entered into the analysis as a classical confounder for brain atrophy. AD, Alzheimer’s disease; NC, normal cognition; PAR, parenchyma.*

The average blood glucose level at each time point was not associated with WMH in the NC group. Glucose levels at 2 h after breakfast and fluctuations in blood glucose levels were associated with brain atrophy. The frequency of hypoglycemia (glucose level ≤ 70 mg/dL) was weakly related to WMH in the temporal lobe. Mild hypoglycemia defined as a low glucose ≤ 80 mg/dL was observed at 4.3% of all SMBG measuring points, and mild hypoglycemia defined as a low glucose ≤ 90 mg/dL was observed at 10.1% of all SMBG measuring points in subjects with NC. However, there were no associations between the frequency of mild hypoglycemia and MR imaging parameters.

### Independent Risk Factors for Regional WMH and Brain Atrophy

To determine whether blood glucose was independently associated with regional WMH and brain atrophy, we conducted multiple regression using a step-wise method (**Table 3**). When WMH was entered as a dependent variable, age, hypertension, HbA1c and daily glucose indices (*p* < 0.10 in **Table 2**) were entered as independent variables. When brain atrophy was entered as a dependent variable, age, HbA1c and daily glucose indices (*p* < 0.10 in **Table 2**) were entered as independent variables. The results showed that elevated blood glucose levels at 2 h after breakfast were independently associated with frontal WMH in the AD group. Blood glucose levels at 2 h after breakfast were inversely associated with PAR volume. We could not identify the independent risk factors for WMH in the temporal lobe in the NC group. Age and blood glucose levels at 2 h after breakfast were independently associated with global brain atrophy in the NC group.

**Table 3:** Independent risk factors for regional WMH and brain atrophy.

	Factors	β	95% CI	*p*-value
**AD subjects**				
WMH in frontal lobe	2 h after breakfast	0.489	(0.002; 0.017)	0.018
PAR	2 h after breakfast	−0.442	(−0.085; −0.003)	0.035
**NC subjects**				
WMH in temporal lobe	N/A		N/A	
PAR	2 h after breakfast	−0.470	(−0.107; −0.030)	0.001
	Age	−0.460	(−0.665; −0.179)	0.001

*Multiple regression analysis with the step-wise method. The dependent variables were the MR imaging parameters. The independent variables were the daily glucose indices (*p* < 0.10 in the previous analysis). Age, hypertension and HbA1c were entered into the analysis as classical confounders for WMH. Age and HbA1c were entered into the analysis as classical confounders for brain atrophy. AD, Alzheimer’s disease; NC, normal cognition; PAR, parenchyma; WMH, white matter hyperintensity.*

## Discussion

The present study revealed that postprandial hyperglycemia was independently associated with frontal WMH in patients with AD. In addition, postprandial hyperglycemia was significantly associated with brain atrophy, regardless of whether cognitive decline was present. Altogether, our findings indicate that postprandial hyperglycemia is associated with WMH in only AD patients, which suggests that AD patients are more susceptible to postprandial hyperglycemia associated with WMH.

To the best of our knowledge, this study was the first to clarify the effects of postprandial hyperglycemia on regional WMH and brain atrophy. In the current study, glucose fluctuations were calculated as diurnal ranges between peaks and nadirs. Glucose levels usually peaked at 2 h after breakfast, and nadirs were observed early in the morning and during inter-prandial periods (Monnier et al., 2007). A large prospective cohort study revealed that postprandial glucose but not fasting glucose was independently associated with AD and VaD (Ohara et al., 2011). This study has shown that diurnal fluctuations in blood glucose are a risk factor for neurodegeneration and cerebrovascular damage. Several studies have obtained conflicting results regarding the associations between glucose indices and WMH. One study found that higher HbA1c levels were associated with WMH (Schneider et al., 2017), while other studies did not find such an association (Exalto et al., 2014; Raffield et al., 2016). These controversies may result from a failure to capture daily glucose variability. HbA1c reflects the average glucose level over the past 4–8 weeks; however, patients with similar HbA1c levels may experience fluctuations in their glucose levels. Our observations implied that measurements of daily glucose variability may better capture the contributions of abnormal glucose metabolism to WMH. Physicians should manage glucose fluctuations carefully, particularly to avoid rapid increases in postprandial glucose levels.

The relationship between postprandial hyperglycemia and WMH was found to be consistent after adjustment for classical confounders, including age and hypertension. Hyperglycemia increases the formation of advanced glycation end-products, which induces oxidative stress and inflammation and exacerbates brain microvascular endothelial function (Tamura and Araki, 2015). However, glucose variability also independently contributes to the development of microvascular complications (Smith-Palmer et al., 2014). Moreover, acute glucose fluctuations are a greater trigger of oxidative stress than sustained hyperglycemia (Monnier et al., 2006). Unfortunately, no data pertaining to oxidative stress levels were available in this study. However, glucose fluctuations and postprandial glucose levels are associated with oxidative stress levels (Ceriello et al., 2002; Monnier et al., 2006). Increased oxidative stress is observed in the diabetic brain (Raza et al., 2015), and the brain is vulnerable to oxidative damage because it consumes high levels of oxygen and has high lipid content and weak antioxidant defenses (Muriach et al., 2014). Furthermore, the brain tissue of patients with AD is exposed to oxidative stress during the course of the disease (Gella and Durany, 2009). Therefore, our observations suggest that postprandial hyperglycemia may induce additional oxidative stress in the AD brain, which is already under increased oxidative stress, and this might be a cause of WMH progression in AD.

Interestingly, we noted an association of postprandial hyperglycemia with WMH in the frontal lobe. Previous study indicated that diabetes patients have greater WMH in the frontal lobe, and WMH volume is associated with reduced cerebral blood flow velocities in the middle cerebral arteries (Novak et al., 2006). More recently, insulin resistance in association with lower cerebral perfusion in the frontal and temporal regions has been proposed (Hoscheidt et al., 2017). Diabetes induces endothelial dysfunction and permeability of the blood-brain barrier, which may affect the regional blood flow and metabolism of the brain (Novak et al., 2006; Kaplar et al., 2009). In line with current literature, our observation suggests that abnormal glucose metabolism might be sensitively associated with cerebral circulation and metabolism, mainly in the frontal lobe.

The association between postprandial hyperglycemia and frontal WMH was observed only in AD. In the current study, patients with AD had a larger global WMH volume, which corresponded to grade 2–3 on the Fazekas scale, than patients with NC, whose WMH volume corresponded to grade 1 (Ogama et al., 2015). WMH volume increased markedly with aging, particularly in the frontal lobe in patients aged 75 years and over who were in the AD/aMCI cohort (Ogama et al., 2016). The pathogenesis of WMH is most likely driven by ischemia resulting from hypertensive small vessel disease, but amyloid angiopathy may also be another underlying cause of extensive WMH (Tomimoto, 2015). More recently, carriage of the apolipoprotein E4 genotype was reported to affect the relationship between glucose alterations and WMH (Livny et al., 2016; Cox et al., 2017). Thus, patients with AD have a variety of pathologies, including small vessel disease, and may be more susceptible to the effects of glucose exacerbation on WMH progression. There was no association between glucose indices and WMH in patients with NC. However, a recent study used the glycoalbumin/HbA1c ratio, a marker of glucose variability, to reveal a significant relationship between these glucose indices and WMH in subjects with NC (Tamura et al., 2017). Additional studies are needed to confirm this association in large populations of elderly patients with diabetes.

In the current study, we noted a weak association between hypoglycemia and temporal WMH in patients with NC; however, whether the two variables were independently associated with each other was less clear. Furthermore, we could not find such an association in patients with AD, who are considered susceptible to glucose metabolism exacerbations. This result may be explained by the study participants’ blood glucose levels being relatively well controlled and severe hypoglycemic events being rarely observed during the study period. Therefore, it was difficult to evaluate the effects of hypoglycemia diagnosed based on the presence or absence of hypoglycemic symptoms on brain abnormalities. The Action to Control Cardiovascular Risk in Diabetes-Memory in Diabetes (ACCORD-MIND) trial found no significant association between symptomatic severe hypoglycemia and abnormal white matter volume (Zhang et al., 2014). We further addressed concerns about repeated mild hypoglycemia in associated with brain abnormalities; however, the results indicated that mild hypoglycemia is not associated with WMH or brain atrophy.

Accumulating evidence indicates that diabetes is associated with global and regional brain atrophy (Falvey et al., 2013; Moran et al., 2013; Hirabayashi et al., 2016; Raffield et al., 2016; Schneider et al., 2017). The results of our study extend current observations and clearly demonstrate that postprandial hyperglycemia is independently associated with global brain atrophy. A previous study indicated that the regional brain atrophy patterns of elderly patients with diabetes resemble those of patients with AD (Moran et al., 2013; Schneider et al., 2017). Regarding its regional distribution, brain atrophy was found in the frontal, temporal, and parietal lobes and the hippocampus, and cortical gray matter loss was more prominent in the left hemisphere than in the right hemisphere (Moran et al., 2013; Schneider et al., 2017). Furthermore, insulin resistance is significantly associated with AD-induced progressive regional atrophy (Willette et al., 2013), and higher glucose levels are significantly associated with an increased risk of dementia, including AD (Crane et al., 2013). Because the association between postprandial hyperglycemia and global brain atrophy was also seen in subjects with NC, we can conclude that accurate glucose control is necessary to prevent brain atrophy.

The present study has several limitations. First, the current study had a cross-sectional design. Therefore, our findings regarding the causal relationship between postprandial hyperglycemia and WMH and brain atrophy should be considered carefully. Second, we examined brain atrophy by parenchymal volume. WMH are associated with white matter atrophy and cortical thinning (Wardlaw et al., 2015). Importantly, WMH in AD patients involves both vascular changes and neurodegenerative mechanisms (Tomimoto, 2015). Thus, it is necessary to separately measure gray and white matter volume to assess the burden of WMH in AD brain. Consequently, it may be more appropriate to estimate regional brain atrophy using voxel-based morphometry by statistical parametric mapping (Adduru et al., 2017). Moreover, although we used a standard 1.5T scanner to examine WMH, high-field MR scanners have greater sensitivity for detecting small WMH (Sicotte et al., 2003; Zwanenburg and van Osch, 2017). Accordingly, future research should use high-field MR scanners to detect subtle WMH. Third, the daily glucose indices measured in this study are not indicators of long-term glucose profiles. However, we investigated daily glucose indices during 8 separate days over a 2-month period to avoid time point deviation in glucose values; thus, our findings may reflect the quality of glucose management over this period. Although this study used SMBG to evaluate glucose levels, CGM might more accurately measure glucose profiles (Bergenstal, 2015). Nonetheless, it is difficult to continuously monitor glucose levels using CGM in patients with AD. Therefore, the use of SMBG might be suitable in studies that include patients with cognitive decline. Finally, the number of study participants was relatively small. However, our sample size was largely comparable to that of a previous study that found that patients with AD-related pathological changes are susceptible to glucose changes associated with the progression of WMH (Livny et al., 2016). Additional longitudinal studies with a large sample size may provide further evidence to confirm our findings.

## Conclusion

In conclusion, postprandial hyperglycemia was found to be independently associated with frontal WMH in patients with AD. In addition, postprandial hyperglycemia is significantly associated with brain atrophy, regardless of the presence of cognitive decline. Thus, our findings suggest that postprandial hyperglycemia is associated with WMH in AD patients but not NC patients, which suggests that AD patients are more susceptible to postprandial hyperglycemia associated with WMH. Our current study provides preliminary results but further longitudinal studies are needed to clarify the effect of glucose management on WMH progression. This study involves a small sample size based on a single dataset, thus confirmation in future longitudinal studies with a larger cohort is required.

## Author Contributions

NO analyzed the data and wrote the manuscript. TS designed the study, analyzed the data, performed the data acquisition, and wrote the manuscript. SK, TT, HT, SS, HM, AS, and MKo performed the data acquisition and contributed to the discussion. SN provided control resources. KT, HU, and MKu reviewed and edited the manuscript.

## Conflict of Interest Statement

The authors declare that the research was conducted in the absence of any commercial or financial relationships that could be construed as a potential conflict of interest.
